# Accuracy of malaria diagnosis by clinical laboratories in Belgium

**DOI:** 10.1186/s12936-019-2731-0

**Published:** 2019-03-28

**Authors:** Laura Loomans, Anali Conesa Botella, Agnes D’hondt, Jacob Verschueren, Dorien Van den Bossche, Marjan Van Esbroeck, Jan Jacobs

**Affiliations:** 1 Department of Clinical Sciences, Institute of Tropical Medicine, Antwerp, Belgium; 2 Department of Microbiology and Immunology, KU Leuven, Louvain, Belgium

**Keywords:** Malaria, Diagnostic performance, Belgium

## Abstract

**Background:**

The Belgian Reference Laboratory for *Plasmodium* offers a free-of-charge reference testing of malaria-positive or doubtful samples to clinical laboratories.

**Methods:**

The final malaria diagnosis from the Reference Laboratory (microscopy, rapid diagnostic tests (RDTs) and *Plasmodium* species-specific PCR) were compared with the final diagnosis from peripheral Belgian laboratories. The Reference Laboratory reports were analysed for all samples submitted between 2013 and 2017. Criteria assessed included the diagnosis of malaria, *Plasmodium* species identification including mixed infections, and in case of *Plasmodium falciparum*, the parasite density and the presence of sexual and asexual stages.

**Results:**

A total of 947 non-duplicate samples were included. Reference testing confirmed 96.3% (893/927) and 90.0% (18/20) samples submitted as positive and negative, respectively, the two missed diagnoses were samples with *Plasmodium ovale* and *Plasmodium malariae*. Submitting laboratories had correctly identified *P. falciparum* in 95.1% (508/534) samples with *P. falciparum* single infection. They had correctly diagnosed the species in 62.9% (95/151) single non-falciparum samples and had reported ‘non-falciparum’ in another 26 (17.2%) samples; most errors occurred among *P. malariae* (n = 8/21, 38.1%) and *P. ovale* (n = 14/51, 27.5%). Only one of the 21 mixed *Plasmodium* species infections had been diagnosed as such by the submitting laboratories; in three of them, *P. falciparum* had been overlooked. Taken single and mixed infections together, *P. falciparum* was diagnosed in 98.6% (546/554) samples. Among 471 single *P. falciparum* samples available for comparison, laboratories had correctly reported parasite densities above 2% in 87.5% (70/80) samples; they had incorrectly reported parasite densities > 2% in an extra 52 (8.9%) samples. Laboratories had correctly reported *P. falciparum* schizonts and gametocytes in 25.6% (11/43) and 56.7% (17/30) samples, respectively.

**Conclusion:**

Diagnostic laboratories in a malaria non-endemic setting provided excellent diagnosis of malaria and *P. falciparum*, reasonably good diagnosis of non-falciparum infections and acceptable calculation of *P. falciparum* parasite density.

## Background

Malaria is a major health problem with 216 million cases and 445,000 deaths worldwide in 2016 [1]. It may be caused by five *Plasmodium* species: *Plasmodium falciparum*, *Plasmodium ovale*, *Plasmodium vivax*, *Plasmodium malariae,* and *Plasmodium knowlesi.* Among them, the most severe with the higher mortality rate is *P. falciparum,* followed by *P. knowlesi* [2].

Currently, there is a risk of malaria transmission in 91 countries and 125 million travellers are at risk every year [3]. As international travel and immigration from endemic zones has increased, there has been an increase in the number of reported cases in non-endemic countries. In Europe 13,000 to 16,000 cases are annually reported, with a facility rate of 2 to 3% [4]. The Study Group on Clinical Parasitology of the European Society for Clinical Microbiology and Infectious Diseases highlights the need for timely and correct diagnosis and refers to Giemsa-stained thick and thin blood films as the reference method [2]. However, due to the low exposure to malaria-positive samples, expertise in diagnosis is thought to be lacking [4, 5].

To support diagnostic laboratories in the diagnosis of malaria, the Belgian Reference Laboratory for *Plasmodium* offers a free-of-charge cross-reference testing of malaria-positive and doubtful samples. Clinical laboratories submit samples together with a request form listing their own diagnosis and the Institute of Tropical Medicine ((ITM), Antwerp, Belgium) returns a report on the day of receipt. In the present study, was aimed to assess the accuracy of each final malaria diagnosis made by submitting laboratories by comparing it to the final diagnosis made by the Reference Laboratory.

## Methods

### Study design

The study compares the malaria diagnosis made by Belgian clinical laboratories for the samples they had submitted voluntarily for cross-reference to the Belgian Reference Laboratory for *Plasmodium* at the ITM, for the period January 2013 to June 2017. The study was designed to compare the final *Plasmodium* diagnosis, not to discuss the different diagnostic methods used.

### Samples

Samples comprised stained and unstained thick and/or thin blood films, a tube of EDTA-anticoagulated blood and a request form with information about patient identity and country of travel or origin, as well as the diagnosis made by the submitting laboratory, including *Plasmodium* species, parasite density, stages, and results of the malaria rapid diagnostic test (RDT).

### Definitions

In case of multiple referrals per patient (e.g., follow-up samples after treatment or new sample in another hospital after transfer of the patient), only the samples from the first referral were considered, unless the time interval between the successive samples was 2 months or more. Data were considered as ‘insufficient data’ if information from the submitting laboratory for both microscopy and RDT results was lacking.

### Reference testing

Reference testing consisted of microscopy, RDTs and *Plasmodium* species-specific PCR. Samples were assessed by an expert microscopist according to World Health Organization (WHO) standards for microscopy, with the exception that the Giemsa staining was done with pH 8.0 instead of pH 7.2 [6]. Presence of *Plasmodium* parasites, species identification and parasite density were assessed, as well as the presence of asexual (trophozoites and schizonts) and sexual (gametocytes) stages and pigment in white blood cells (WBC). Two RDTs were carried out: Carestart™ (Access Bio, Somerset, USA) Malaria Pf (pLDH)/Pan (pLDH) and SD Bioline (Abbott Laboratories, Abbott Park, IL, USA) FK60 Malaria Ag P.f. (HRP-2)/Pan (pLDH). Additionally, SD Bioline FK80 P.f. (HRP-2)/P.v. (pLDH) detecting *P. falciparum* and *P. vivax*-specific parasite lactate dehydrogenase was used in case of microscopic identification of *P. vivax* or *P. ovale*. Parasite densities were estimated by counting asexual parasites against 200 WBC in thick blood films and using the actual WBC count of the patient for calculation or, when not available, a standard value of 8000 WBC/μl [6]. All positive and doubtful results were verified by a second microscopist. Expert opinion of a clinical microbiologist was invoked in case of aberrant results. Results were reported the same day as receipt of the sample. Next, real-time PCR (four-primer available) was done on all malaria positive samples within a week [7].

### Database

Patient data and the final diagnosis made by the Reference Laboratory were extracted from the ITM Reference Laboratory Information System (LAB400; Cegeka NV Hasselt, Belgium) into an Excel database (Microsoft Office 2013, Santa Rosa, CA, USA). Data about the diagnosis recorded by submitting laboratories were manually encoded. Incoherent data were verified against the original submission form. The database used for the analysis was coded and did not contain any patient identity or confidential information.

### Data analysis

Data were analysed with Excel and Graphpad Prism Version 5.01 (Graphpad Software, CA, USA). Diagnoses made by submitting laboratories were compared to those made by the Reference Laboratory. Criteria assessed included (i) diagnosis of malaria; (ii) *Plasmodium* species identification including mixed infections; and, (iii) in case of *P. falciparum*, the parasite density and the presence of sexual and asexual stages. As most laboratories reported parasite density as % of infected red blood cells (RBC), the reference parasite density expressed per µl was converted to % of infected RBC by dividing by 50,000. The reported parasite density was considered acceptable when the difference with the reference was within 1 log; in addition, the agreement for the parasite density threshold of 2% for severe malaria infection was assessed.

## Results

### Study population and sample collection

**Fig. 1:**
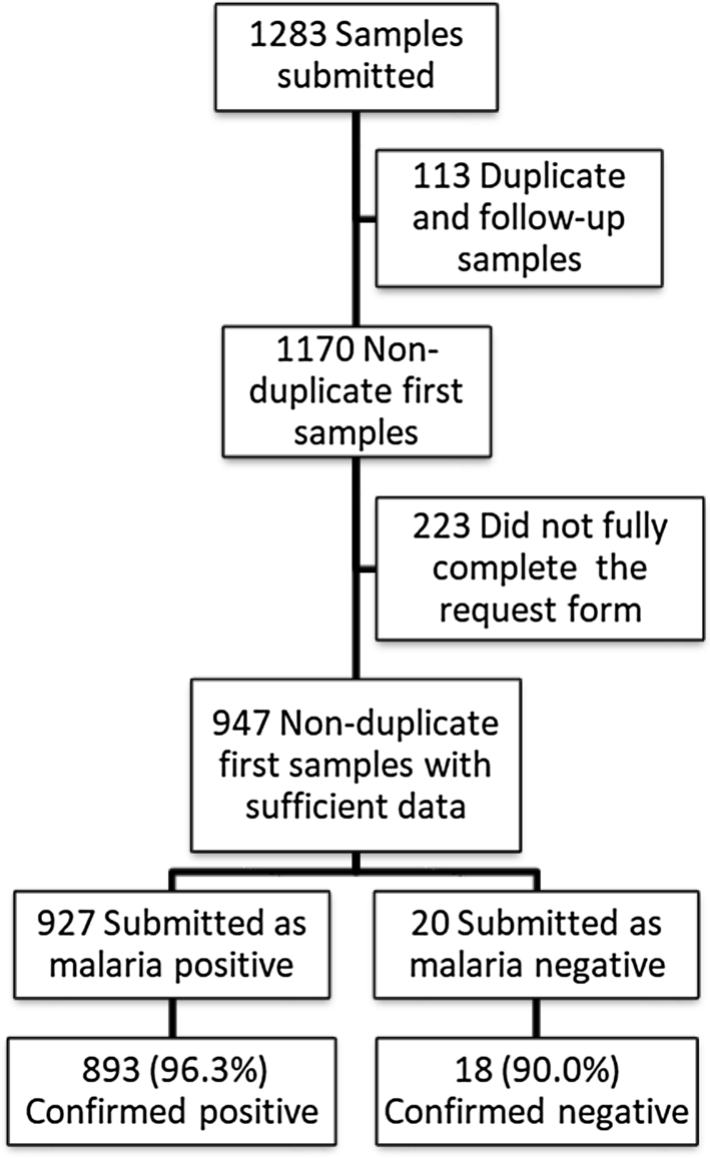
Breakdown of samples submitted by diagnostic laboratories in Belgium to the reference laboratory for confirmation of malaria diagnosis (2013–2017)

Out of 1283 submitted samples, there were 1170 non-duplicate first samples, of which 947 (80.9%) had enough data for analysis (Fig. 1). Median age was 36 years (min–max: 1–84 years), 146 (15.4%) were younger than 18 years old, male-to-female ratio was 1.8 (610/337). Region of travel or origin (data available for 814 samples) was Africa (89.8% mainly from the Democratic Republic of the Congo, Ghana and Nigeria), followed by the Eastern Mediterranean region (7.0%), Southeast Asia (2.0%), the Western Pacific region (0.6%), and continental America (0.5%). Upon reference testing, 893/947 (94.3%) samples tested as malaria positive, submitted as 27 (3.0%) mixed infections and 857 single species infections, including *P. falciparum* 649/893 (72.7%), *P. vivax* 96/893 (10.8%), *P. ovale* 77/893 (8.6%), and *P. malariae* 35/893 (3.9%). The 9 remaining samples were reported as positive without further species reported. The median parasite density for *P. falciparum* single infections quantified by reference testing was 7110/µl (range 1–1,489,632/µl).

Submitting laboratories (n = 119) represented 69.2% out of 172 laboratories subscribing to the proficiency testing for malaria diagnosis in 2013 [8], and submitted results obtained by both microscopy and RDTs (n = 761, 80.4%), microscopy alone (n = 149, 15.7%), and RDT alone (n = 37, 3.9%). The number of samples submitted per year varied from 163 in 2013 to 257 in 2016, with a mean of 209 per year, with highest numbers submitted during the northern hemisphere summer holidays (June to September).

### Accuracy of results obtained by reference testing: diagnosis of malaria

A total of 927 (97.9%) samples were submitted as malaria-positive, the remaining 20 (2.1%) as malaria-negative (Fig. 1). Samples submitted as malaria-positive were confirmed by reference testing in 893/927 (96.3%) cases. Of the remaining 34 (3.7%) non-confirmed malaria-positive samples, 25 had been submitted with a comment expressing doubts about the diagnosis, the other 9 (1.0%) had been misdiagnosed by microscopy and RDT (n = 2; *P. falciparum* and *Plasmodium* non-falciparum, respectively) and by RDT alone (n = 7; data about antigen test lines were not recorded). Of the 20 samples submitted as malaria-negative, 18 (90.0%) were confirmed as negative; the 2 remaining samples were diagnosed by reference testing as *P. ovale* (parasite density 6583/µl) and *P. malariae* (the sample received was not appropriate for quantification of parasite density). The number of positive samples that were confirmed as positive increased over time: 94.2% samples were confirmed positive in 2013, 95.8% in 2014, 96.2% in 2015, and 98.0% in 2016 (p = 0.040; Chi Square test).

### *Plasmodium* species identification in single and mixed infections

Laboratories had reported *Plasmodium* species identification in 706/893 (79.1%) malaria positive samples (Table 1).

**Table 1:** Comparison of *Plasmodium* species identifications made by the submitting clinical laboratories matched with those obtained at reference testing, for 706 samples for which data from the submitting laboratories were available

Species identification	Reference laboratory							
Submitting laboratories	Pf	Pv	Po	Pm	PfPv	PfPo	PfPm	PoPm
Pf	508^a^	2		1	2	9	5	
Pv	1^b^	64^a^	8	1				
Po	3^b^	3	22^a^	5		1^b^		
Pm		2	3	9^a^			1^b^	
Pfmx	9						1	
PfPo	7		1					
PfPv	2							
PfPm	4							
PoPm			2	1				
PvPoPm			1					
Pnon-f		8	14	4		1^b^		1
Total	534	79	51	21	2	11	7	1

Pf, *P. falciparum*; Pv*, P. vivax*; Po, *P. ovale*; Pm, *P. malariae*; Pfmx, mixed infection including *P. falciparum*; Pnon-f, *P. non*-*falciparum*

Codes: ^a^ denotes correct identification by the diagnostic laboratory, ^b^ denotes missed diagnosis of *P. falciparum*

### Single infections

Among 534 samples diagnosed as *P. falciparum* single infection at reference testing, submitting laboratories had correctly identified *P. falciparum* in 508 (95.1%) samples (Table 1). An additional 22 (4.1%) samples had been erroneously identified as mixed infection but with *P. falciparum* among the species present; they had misidentified *P. falciparum* in four (0.7%) samples: *P. ovale* (n = 3) and *P. vivax* (n = 1). Reference testing further diagnosed 151 samples as single infections by *P. vivax*, *P. ovale*, or *P. malariae*: laboratories had correctly diagnosed the reference species in 95 (62.9%) samples and had reported non-falciparum in another 26 (17.2%) samples; the remaining 30 (19.9%) samples had been incorrectly identified, among which were three misdiagnoses as *P. falciparum*. Errors were highest among *P. malariae* (n = 8/21, 38.1%) and *P. ovale* (n = 15/51, 29.4%).

### Mixed infections

Among the 21 samples with mixed *Plasmodium* species infections identified at reference testing, submitting laboratories had diagnosed only one as a mixed infection. Most (20) of these samples contained *P. falciparum*, which had been correctly reported by the submitting laboratories in 17/20 (85%) samples. In three other samples, the presence of *P. falciparum* had been overlooked. Taken single and mixed infections together, *P. falciparum* was correctly diagnosed in 98.6% (547/554) samples.

### *Plasmodium falciparum*: quantification of parasite density and identification of stages

For 485/534 (90.8%) single *P. falciparum* samples, submitting laboratories had reported parasite densities, mostly (n = 469, 96.7%) expressed as % of infected RBC. In 13 cases, only descriptive terms were used (rare, very low, only 1 parasite seen) and in one case, the reference laboratory could not reliably assess the parasite density. Among 471 *P. falciparum*-positive samples available for comparison, 374 (79.4%) of densities recorded by the submitting laboratory were within 1 log of the reference value. Eighty (17.0%) *P. falciparum* samples had a parasite density > 2%; for 70 of them (87.5%), laboratories had also correctly reported values above 2%. Among the samples incorrectly reported as < 2% (n = 10), parasite densities reported were < 0.5% for four samples. Conversely, laboratories had reported parasite densities > 2% in an extra 52 (8.9%) samples compared to reference testing, with 16 among them counted as < 0.5% by reference testing.

Laboratories providing parasite density had also reported *P. falciparum* stages (Table 2). Of 43 samples containing schizonts, approximately one-quarter (n = 11, 25.6%) had been reported by the submitting laboratory, while gametocytes had been reported in 56.7% (n = 17/30) of samples.

**Table 2:** Comparison of the stages of *Plasmodium falciparum* reported by the submitting laboratories matched with those obtained at reference testing, for 485 samples for which data were available

Stages identification	Reference laboratory				
Submitting laboratories	t	tg	ts	tgs	g
t	390^a^	11	29^b^	2^b^	
tg	2	11^a^	1^b^		
ts	10		8^a^		
tgs	1	1	2	1^a^	1^d^
g	3^c^	1^c^			2^a^
s	1				
Total	407	24	40	3	3

*t* trophozoite, *g* gametocyte, *s* schizont

Codes ^a^ denote correct identification of all stages, ^b^ denote schizonts missed, ^c^ denote only gametocytes reported in sample containing trophozoite, ^d^ denote trophozoites and schizonts reported in samples containing only gametocytes

## Discussion

Malaria diagnosis in non-endemic settings faces many difficulties among which are timely sample preparation and staining, low parasite densities, altered parasite morphology caused by chemoprophylaxis or empiric therapy, but most of all inexperience in malaria microscopy [4, 9–11]. Challenges are even higher for initial diagnosis during out-of-office hours, when competent microscopists may not be available [12, 13].

Malaria diagnosis made by submitting laboratories was confirmed by reference testing in 96.3% of samples and submitting laboratories had missed *P. falciparum* in only 0.7% of single *P. falciparum* samples. Given its specific treatment and potential fatal complications, reporting the presence of *P. falciparum* is of crucial importance. Accuracy of *P. falciparum* diagnosis was lower for mixed *Plasmodium* species infections (80.0%); moreover, all but one (20/21) mixed *Plasmodium* infections diagnosed at reference testing had been overlooked as mixed infections by submitting laboratories. As shown in this study, mixed *Plasmodium* infections are rare events but they mostly contain *P. falciparum* and should be treated accordingly [14].

Quantifying and reporting parasite density is part of standard malaria diagnosis as it reflects the severity of the *P. falciparum* infection and it is used for treatment follow-up. In non-immune travellers, parasite densities exceeding 100,000/µl (equivalent to > 2% of RBC infected) point to an increased risk for complications and a need for intravenous treatment [14]. Over 90% of laboratories had quantified parasite densities for *P. falciparum* and accuracy was satisfactory. It should be noted, however, that the criterion for acceptability was set broad, in line with the high variation of parasite counting, particularly at low parasite densities [15]. Most laboratories used the ‘% of infected red blood cells’ for counting and expressing parasite density, which is well understood by the attending clinician but less convenient for expressing low parasite densities. Although not requested as part of routine diagnosis, these laboratories had also reported stages in the case of *P. falciparum*. Almost three-quarters and nearly half of them had not observed *P. falciparum* schizonts and gametocytes, respectively. The *P. falciparum* schizont stage is usually restricted to the organ capillaries, and its presence in peripheral blood may alert to an increased risk for complications [14]. The presence of *P. falciparum* gametocytes together with trophozoites indicates a longer standing (> 7–10 days) infection, whereas the unique presence of gametocytes after treatment is a regular finding and not a sign of drug resistance [9].

The identification of non-falciparum species was lower than of *P. falciparum* species. However, a good performance was noted for the differentiation of non-falciparum species, with most errors observed for *P. malariae* and *P. ovale*. As for *P. falciparum*, errors tended to occur more frequently among mixed *versus* single infections. The interest of non-falciparum species differentiation is that *P. vivax* and *P. ovale* form hypnozoites (dormant stages) in the liver that can cause relapses and need primaquine treatment [16]. Although this treatment is best given in conjunction with the blood-stage treatment, there is less of an emergency and the diagnosis of non-falciparum malaria (implying exclusion of the presence of *P. falciparum*) is acceptable pending further species differentiation in a reference laboratory.

Most data for comparison are derived from External Quality Assessments (EQA), reported from the UK (1986–2001) [17], Canada (1995–1997) [18], USA (1999–2008) [11], and Hong Kong (2002–2006) [10]. Unlike in the present study, they generally submit panels with few samples and results are expressed as % of participants, not samples. These EQAs consistently reported difficulties and shortcomings in the detection of *P. falciparum*, the diagnosis of mixed species infections, the estimation of the *P. falciparum* parasite density, and differentiation between the non-falciparum species. Failures of *P. falciparum* diagnoses were observed among 11–27.3% [11, 18]. Conversely, 7% of participants misidentified non-falciparum species as *P. falciparum* and 2–11.3% of participants reported the presence of malaria parasites on a normal blood film [11, 18]. Likewise, species identification of the non-falciparum species was moderately accurate, with 22.5, 21.7 and 100%, respectively, for *P. malariae*, *P. vivax* and *P. ovale* [11]. Mixed infections raised problems too, with a very low accuracy of identification of both *Plasmodium* species (13–27% in one study [17]). Not quantifying *P. falciparum* density (≥ 25% of the participants) was another consistent finding [11, 17, 18] and when done, errors were made by 13–39% of participants with a tendency for overestimation [17, 19]. Among the explanations evoked were counting multiple trophozoites in a single RBC, underestimating the total amount of counted RBC, as well as counting gametocytes and non-falciparum parasites in mixed infections, and unlike other errors, quantifying the parasite density did not improve over time [19].

Few studies assessed the accuracy of routine malaria diagnosis of clinical laboratories. Two studies had a similar design as the present study, i.e, they cross-checked routine samples submitted to reference laboratories in the UK and Hong Kong [10, 20]. In addition to numerous technical shortcomings in thick blood film preparation and staining, the UK study showed a failure rate of 21% for the diagnosis of *P. falciparum* and a poor accuracy for species identification of *P. ovale*. The Hong Kong study assessed both EQA and cross-checking samples with similar findings as the UK study; in addition, it showed a poorer performance for the cross-checking samples as compared to the EQA samples. A third study assessed routine malaria diagnosis among a cohort of African refugees in Canada in 2000. Main findings were that 5/20 malaria diagnoses were not confirmed by reference testing and that parasite density were reported in only 5/20 samples [21].

Interestingly, laboratories in this study performed much better than EQAs. This is surprising as EQAs reflect the best rather than day-to-day performance [10]. Several reasons may explain this difference. First, most previous studies date from at least a decade ago, when international diagnostic guidelines, online training [15] and procedures were not yet available or widespread. Second, all laboratories in the present study were subscribing to the Belgian EQA provider, which has offered many didactic sessions about malaria microscopy over recent years [22]. Third, although the present study was not designed to compare different diagnostic procedures, it was assumed that RDTs have been a valid adjunct to diagnosis. Indeed, over 80% of diagnoses were made by both microscopy and RDT. Use of RDTs among clinical laboratories in Belgium is higher than previously reported in the UK and the USA [23, 24]. Malaria RDTs have an excellent sensitivity for *P. falciparum* and are accurate to rule-in and rule-out the presence of *P. falciparum*. Recently marketed RDTs designed to detect *P. vivax* have a 95% sensitivity (although dependent on parasite density) to diagnose this species, but RDTs still perform poorly (< 50% sensitivity) for *P. ovale* and *P. malariae* [25, 26]. Given their limitations, malaria RDTs are recommended as an adjunct and not as a replacement of microscopy for the diagnosis of malaria in non-endemic settings [2, 15, 24, 25]. Finally, in this study it is believed that reference testing by ITM may contribute to good practice as the request form asks for data, such as species identification and parasite densities [27], and as the result of reference testing is reported timely, thereby providing contextual feedback.

In the practice of ITM reference testing and as shown in this study, laboratories nearly exclusively submitted malaria-positive samples and only a few doubtful or negative samples. Therefore, the present study did not allow for tracing missed or delayed diagnosis of malaria. Among the other strengths of the study, are the high number and consistent submission of samples over a 4-year period and high representation among clinical laboratories.

Of note, there were no *P. knowlesi*-infected samples in the present study. Given its potential fatal complications, and similarly to *P. falciparum*, species recognition of *P. knowlesi* and quantification of its parasite density is imperative [28]. Current RDTs are not reliable for detecting *P. knowlesi* [15].

## Conclusion

This study showed that diagnostic laboratories in malaria non-endemic settings provided excellent diagnosis of malaria and especially the detection of *P. falciparum*. They performed reasonably well in determining *P. falciparum* parasite density as well as in the diagnosis of non-falciparum species, but fell short in detection of *P. falciparum* schizonts and gametocytes. The results of this study show a very good performance of malaria diagnosis compared to previous EQA reports from non-endemic settings.

## Abbreviations

EDTA: ethylenediaminetetraacetic acid
EQA: External Quality Assessments
HRP-2: histidine rich protein-2
ITM: Institute of Tropical Medicine
PCR: polymerase chain reaction
pLDH: *Plasmodium* lactate dehydrogenase
RDT: rapid diagnostic test
RBC: red blood cells
WBC: white blood cells
WHO: World Health Organization
